# Machine learning identification of EEG features predicting working memory performance in schizophrenia and healthy adults

**DOI:** 10.1186/s40810-016-0017-0

**Published:** 2016-02-11

**Authors:** Jason K. Johannesen, Jinbo Bi, Ruhua Jiang, Joshua G. Kenney, Chi-Ming A. Chen

**Affiliations:** 1 VA Connecticut Healthcare System, Psychology Service, 116-B, 950 Campbell Ave, West Haven, CT 06516, USA; 2 Psychiatry, Yale University School of Medicine, New Haven, CT, USA; 3 Computer Science and Engineering, University of Connecticut, Storrs, CT, USA; 4 Psychological Sciences, University of Connecticut, Storrs, CT, USA

**Keywords:** EEG, Gamma frequency, Support vector machine (SVM), Machine learning, Sternberg task, Working memory, Schizophrenia

## Abstract

**Background:**

With millisecond-level resolution, electroencephalographic (EEG) recording provides a sensitive tool to assay neural dynamics of human cognition. However, selection of EEG features used to answer experimental questions is typically determined *a priori*. The utility of machine learning was investigated as a computational framework for extracting the most relevant features from EEG data empirically.

**Methods:**

Schizophrenia (SZ; *n* = 40) and healthy community (HC; *n* = 12) subjects completed a Sternberg Working Memory Task (SWMT) during EEG recording. EEG was analyzed to extract 5 *frequency components* (theta1, theta2, alpha, beta, gamma) at 4 *processing stages* (baseline, encoding, retention, retrieval) and 3 scalp sites (frontal-Fz, central-Cz, occipital-Oz) separately for correctly and incorrectly answered trials. The 1-norm support vector machine (SVM) method was used to build EEG classifiers of SWMT trial accuracy (correct vs. incorrect; Model 1) and diagnosis (HC vs. SZ; Model 2). External validity of SVM models was examined in relation to neuropsychological test performance and diagnostic classification using conventional regression-based analyses.

**Results:**

SWMT performance was significantly reduced in SZ (*p* < .001). Model 1 correctly classified trial accuracy at 84 % in HC, and at 74 % when cross-validated in SZ data. Frontal gamma at encoding and central theta at retention provided highest weightings, accounting for 76 % of variance in SWMT scores and 42 % variance in neuropsychological test performance across samples. Model 2 identified frontal theta at baseline and frontal alpha during retrieval as primary classifiers of diagnosis, providing 87 % classification accuracy as a discriminant function.

**Conclusions:**

EEG features derived by SVM are consistent with literature reports of gamma’s role in memory encoding, engagement of theta during memory retention, and elevated resting low-frequency activity in schizophrenia. Tests of model performance and cross-validation support the stability and generalizability of results, and utility of SVM as an analytic approach for EEG feature selection.

## Background

Electroencephalographic (EEG) recording, when combined with experimental tasks, can provide powerful methodology for studying neural dynamics of human cognition. EEG data is dimensional and complex, based on a time series of events sampled with high temporal resolution and distributed spatially across multiple scalp locations. Given that research-grade EEG systems are capable of sampling at 1000 samples per second and higher, a simple 10-min experiment could feasibly produce 600,000 discrete data points per channel of acquired data even before considering spatial characteristics or signal extraction methods (e.g., amplitude, spectral power, coherence) that further add to possible number of variables produced. Analysis of such data requires many decisions about the time points and signal extraction methods used to best characterize the psychophysiological phenomena under investigation. Without standardized procedures for EEG experimentation or data extraction across laboratories, how are these decisions to be made? It seems that in most cases, investigators defer to methods of prior studies for guidance on new studies. While this approach may provide important replication of prior results and incrementally advance knowledge, it may also limit EEG analyses to a relatively small portion of the data collected, overlook important features of data not previously discovered, and constrains science to a confirmatory and deductive, rather than data inductive, position.

The primary measure of EEG activity used in psychophysiological research is the event-related potential (ERP). ERPs are defined by stereotyped patterns of voltage change time-locked to stimulus events and are quantified by peak amplitudes measured in averaged waveforms. ERP analysis may therefore focus narrowly on a time window containing a specific peak and leave a large portion of the EEG record discarded from further analysis. However, in addition to the event-related activity driven exogenously by stimulus events, these data contain neural activity generated endogenously that is not captured by averaged waveforms, as well as activity during pre-stimulus and intertrial intervals that may reveal important differences in how the brain prepares for and carries out task-related processes. The importance of pre-stimulus activity, for example, is illustrated in work relating the amplitude of ERPs to resting EEG recorded in a passive state prior to the experiment [1] and in demonstrations of how ERP amplitudes can be altered experimentally by modulating pre-stimulus activity through non-invasive neural stimulation [2]. Accordingly, individual differences in task-related ERP measures, as well as group-wise differences, could be influenced by features of neural oscillatory activity that are inadvertently excluded from conventional ERP analysis. Increased use of time-frequency analysis over the past 10 years [3, 4], and associated measures of coherence and phase synchrony, further extend the range of features that can be extracted from standard ERP experiments and the number of variables that can potentially be submitted to statistical analysis. Given the many sources on information that can be gleaned through various signal processing approaches, there is increased need for computational frameworks capable of mining large datasets to identify features most relevant to questions asked of EEG data.

Machine learning encompasses a body of statistical approaches that can be used to discover knowledge from data through mathematical modeling, wherein pattern recognition is optimized by allowing the program to adjust actions accordingly to new information. Machine learning methods are becoming more commonly used in medical sciences, outperforming classical regression approaches when applied to prediction and diagnostic classification decisions [5, 6]. The Support Vector Machine (SVM) approach, in particular, has proven useful for clinical classification problems based on brain imaging data [7]. SVM provides individual-level classification and, therefore, can be applied to questions pertaining to diagnosis, prediction of treatment response, and progression to illness based on preclinical indicators. Furthermore, because SVM is inherently multivariate, it is an appropriate method for separating unique from redundant sources of variance in spatially distributed, yet variably dependent, patterns of brain activity.

As a method of EEG feature selection, SVM could provide a powerful tool for reducing large data arrays of scalp locations, frequency bands, and temporal windows to those most pertinent to a classification question. For clinical purposes, this approach could be used to build classifiers of known diagnostic categories based on latent patterns of EEG activity, to refine classifiers iteratively through cross-validation, and ultimately to apply validated classifiers to new clinical samples. In experimental research, these methods can be used to identify the EEG features most related to task behavior, thereby allowing the researcher to empirically develop neural models of human behavior without *a priori* knowledge of task-related activity.

The current study aimed to demonstrate the utility of SVM as a data inductive solution for EEG feature selection. The sample consisted of individuals with schizophrenia and healthy community members who performed a Sternberg working memory task during EEG recording. The Sternberg task can be analyzed over stages of encoding, retaining, and retrieving information from short-term memory, each involving different sources and components of brain activity, with all contributing to successful task performance. Therefore, multiple spectral-frequency, temporal, and spatial characteristics must be considered simultaneously in order to answer questions about patterns of optimal task-related brain activity and differences in schizophrenia. Questions such as this seem most amenable to empirical approaches of feature selection as (a) the number of variables that could be conceivably extracted from these data far exceed the number of comparisons that would be advisable if tested independently, and (b) the dynamics of EEG, involving changes and interactions in sources of brain activity that co-vary with individual differences in task performance, can only be resolved in multivariate space where hierarchical relationships within and between features are compared over repeated observations. SVM may provide an appropriate, albeit novel, data reduction and classification approach for this type of analytic problem.

Using a supervised learning approach, given that information about task performance and diagnostic group membership is known, what EEG features would SVM be expected to identify? Working memory is a core domain of neurocognitive impairment in individuals with schizophrenia, found across various task versions administered in auditory and visual modalities [8, 9]. Working memory requires network-level activation and coordination of neural activity between pre-frontal cortical and cortical association areas involved in sensory and attentional processes [10–12]. The cortical distribution of neural activity during working memory performance has been studied extensively using EEG recording [13–15], demonstrating that optimal behavioral performance can be predicted on the basis of neural dynamics [16, 17]. Although these interrelations are complex, and may interact differently depending on memory load and individual differences in performance, task-related changes in theta, alpha, and gamma band spectral power have been consistently reported [18]. Theta band (e.g., 4–8 Hz) activity is associated with hippocampal-cortical communication during encoding [19] and increases with higher memory load [13]. In a model based on the interrelationship of theta and alpha, performance is suggested to be optimal when pre-trial baseline EEG contains low tonic theta power but high phasic alpha power, and when encoding is accompanied by event-related increases in theta band and reductions in alpha band power [17]. A shift to alpha (e.g., 8–12 Hz) is then associated with subsequent memory retention and retrieval processes [14] involving thalamo-cortical networks [20]. Gamma band (e.g., > 30 Hz) activity is generally associated with integrative multi-modal sensory processes and, in memory tasks, appears to couple in-phase with theta [21]. As with theta, gamma band power is normally increased with higher memory load [22, 23]. While related in phase, neural activity in gamma and theta bands are associated with distinct functional roles in memory processing, with gamma supporting short-term maintenance and theta supporting the organization of sequentially ordered information into memory [18]. Importantly, while gamma band power increases may indicate the recruitment of additional cognitive resources required to meet higher task demands, individuals with schizophrenia appear to have a limited capacity to modulate gamma activity in this way [24, 25].

In addition to features embedded in task-related EEG, it is also important to consider the possibility that neural activity unrelated to demands of the task, but perhaps reflecting traits of illness, can also affect performance in schizophrenia. For instance, resting state EEG in schizophrenia is commonly characterized by abnormal elevations in theta and alpha, which persist during experimental conditions where suppression of this activity normally occurs [26]. Based on the previously described neural dynamics model of memory [17], high levels of tonic (i.e., task independent) theta and failure to down regulate alpha during encoding would predictably result in impaired memory function. Taken together, these findings provide basis for predicting that differences in EEG activity during Sternberg task performance will be characterized by elevated low-frequency activity at the pre-stimulus baseline period and by lower levels of event-related theta and gamma spectral power during encoding in schizophrenia. Alternatively, optimal performance should be predicted by higher levels of theta and gamma during encoding, and alpha activity at the retrieval stage. Given these predictions, the primary question pertaining to SVM-based analysis is whether these same features emerge as most critical to Sternberg performance and diagnostic differences when tested within a relatively large array (*n* = 60) of EEG features.

## Method

### Participants

Forty individuals meeting DSM-IV-TR criteria for schizophrenia (SZ) and 12 healthy comparison (HC) participants were enrolled in a registered clinical trial (identifier: NCT00923078, https://clinicaltrials.gov/) at time of this analysis. For purposes of the current analysis, only data collected at study intake will be presented. The study was conducted under oversight of VA Connecticut Healthcare System (VACHS) Human Studies Subcommittee (HHS protocol # 01245) and Yale University Human Investigation Committee (HIC protocol # 1003006388) institutional review boards. All participants provided written informed consent prior to initiating any study procedures and were compensated $75 for data collected at study intake assessment. Recruitment of HC participants was conducted according to match (age, gender, race) with SZ participants. Sample descriptive statistics are presented in Table 1.

Inclusion was limited to individuals aged 18 and 70, native English speaking, with stable housing for minimum of 30 days. In addition, SZ sample members had minimum of 30 days since discharge from last hospitalization, 30 days since last change in psychiatric medications, and were receiving mental health services through VACHS or Yale affiliated outpatient facilities. Individuals were excluded based on current (past 30 days) diagnosis of alcohol or substance abuse disorders, history of brain trauma or neurological disease, mental retardation or premorbid intelligence ≤ 70, and auditory or visual impairment that would interfere with study procedures. In addition, any current or past DSM-IV Axis I diagnosis was exclusionary for HC sample enrollment.

### Clinical assessment measures

All participants underwent a clinical interview to obtain treatment, substance use, medical, legal, employment, and psychosocial background information. Diagnosis of SZ sample participants was confirmed using the Structured Clinical Interview for DSM-IVTR (SCID-I/P; [27]), administered by a licensed clinical psychologist. The Mini International Neuropsychiatric Interview (M.I.N.I; [28]) was administered to healthy volunteers to screen for psychiatric conditions that would be exclusionary. The Wechsler Test of Adult Reading (WTAR; [29]) was administered to all participants to obtain an estimate of premorbid intellectual endowment and the MATRICS Consensus Cognitive Battery (MCCB; [30]) was used to test current cognitive ability across multiple domains. Age- and gender-corrected t-scores for MCCB Working Memory Composite and Continuous Performance Test–Identical pairs (CPT-IP) subtest were used in the current analysis to cross-validate SVM-derived models of EEG activity related to working memory.

### EEG data collection procedures

Participants were seated in front of a 24” LCD monitor (1920×1200 pixels, 75 Hz refresh rate) at a viewing distance of 100 cm in a dimly lit room. EEG was recorded using a 64-channel BioSemi ActiveTwo (BioSemi B.V., Amsterdam, Netherlands) bio-amplifier and electrode system with sensors located according to the 10–20 system. Additional electrodes were placed bilaterally at mastoids (reference), the outer canthi of both eyes (horizontal electrooculogram; HEOG), and above and below the right orbit (vertical electrooculogram; VEOG). Continuous EEG was monitored online in ActiView V6.05 and acquired at a 1024 Hz sampling rate with a bandpass filter setting of 0.16–100 Hz. The Sternberg task was administered using NBS Presentation software (Neurobehavioral Systems, Inc., Albany, CA), with behavioral responses captured using two buttons of a Cedrus RB-834 response pad (Cedrus Corporation, San Pedro, CA). Total EEG set up time was approximately 30 min, and the Sternberg task was administered in three blocks of interspersed between blocks of two additional auditory ERP tasks (not included in current report).

### Sternberg working memory task

A version of the Sternberg working memory task (SWMT), modified from Raghavachari et al. [31], was used in the present study. Stimuli consisted of sequentially presented letters (200 ms duration, 1200 ms ISI), spanning sets of 4–8 letters each, randomly generated from an array of 12 letters. For each trial the stimulus set was followed by a 3200 ms retention period that terminated with a response probe letter. Participants were instructed to press one of two response pad buttons, using right or left index finger, to indicate whether the probe letter was or was not presented in the preceding set. The response probe remained present for the duration of the response window, up to 3500 ms, and terminated at time of button press. Auditory feedback was given to indicate correct, incorrect, or time-out (after 2000 ms) on each trial. Feedback was followed by 1000 ms of black screen and a fixation “+” cross for another 1000 ms preceding the first stimuli of the next set. A total of 90 trials was administered over three blocks of 30 trials, each block lasting approximately 8 mins.

### EEG signal processing

**Fig. 1:**
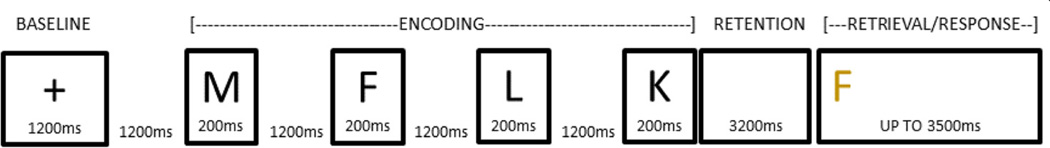
Example of Sternberg Working Memory Task (SWMT) trial depicting span of 4 items and time spans of pre-stimulus baseline, encoding, retention, and retrieval stages. Span ranged from 4–8 items, with span width and items selected randomly on a trial by trial basis

Data analysis was conducted using BrainVision Analyzer software v2.0 (Brain Products, Munich, Germany). SWMT EEG data was re-referenced offline to the average mastoid, broadband filtered from 1–70 Hz (12 dB/oct) with a notch filter at 60 Hz, and segmented according to four stages of processing (Fig. 1); pre-stimulus baseline (500 – 1200 ms relative to fixation), encoding (−200 – 8000 ms relative to fixation), retention (−3400 – 800 ms relative to probe), and retrieval (−200 – 800 ms relative to probe). The analysis window selected for the encoding stage spanned the first 5 letters (or all 4 when span = 4) of each trial. This window was selected to optimize the amount of information that could be consistently extracted across trials varying in length based on span.

Following segmentation, ocular artifact correction was applied [32] and segments containing activity ±75 μV at electrodes Fz, Cz, and Oz were excluded. Time-frequency extraction was applied to single trial data using Morlet continuous wavelet transform (parameter c = 3.8) over 20 frequency steps from 4–50 Hz. Data at encoding and retrieval stages was averaged to extract event-related spectral perturbations (ERSP), elicited in response letter memory and probe stimuli, respectively. Encoding stage frequency extraction was baseline normalized to a window of −200 to −50 ms relative to fixation cross, while retrieval was normalized to a window of −200 to −50 ms relative to response probe onset. The same wavelet transform was applied to EEG data at pre-stimulus baseline and retention stages without normalization. Time-frequency data was output in the form of squared wavelet coefficients (μV^2^) binned and averaged according to response accuracy (correct vs. incorrect), and exported in five frequency bands at each of the four stages of processing: Theta 1 (θ_1_), centered at 4.00 Hz (range: 3.12 – 4.88); Theta 2 (θ_2_), centered at 6.42 Hz (range: 5.01 – 7.83); Alpha (α), centered at 11.26 Hz (range: 8.79 – 13.73); Beta (β), centered at 18.53 Hz (range: 14.46 – 22.59); Gamma (γ), centered at 40.32 Hz (range: 31.48 – 49.16). Time-frequency values were exported for statistical analysis based in the following windows: pre-stimulus baseline (500 – 1200 ms relative to fixation); encoding (1000 – 7000 ms relative to fixation); retention (−3000 − 0 ms relative to probe); and retrieval (0 – 600 ms relative to probe). All statistical analyses were conducted on spectral power measured at three midline electrode locations: Frontal (Fz), Central (Cz), and Occipital (Oz).

### Machine learning feature selection

From a machine learning point of view, our analysis is a variable selection problem that aimed to identify the EEG features most relevant to SWMT performance and diagnostic group differences. Variable selection methods are often divided along two lines: filter and wrapper methods [33]. The filter approach of selecting variables serves as a preprocessing step to the model construction. The main disadvantage of the filter approach is that it ignores the effects of the selected variable subset on the performance of the classification algorithm. The wrapper method searches the optimal variable subsets using the estimated classification accuracy, as the measure of *goodness*, when the subset of variables is used in classification. Thus, the variable selection is being “wrapped around” a particular classification algorithm. Wrapper methods typically outperform filter methods [34].

For the current analysis variable selection was conducted using a wrapper method that is wrapped around the so-called 1-norm SVM [35]. SVM is a supervised learning method which has the ability to weigh input features according to their relevance to the classification target, as determined through the learning process. Most SVMs, including the one implemented in this study, construct a linear classifier that predicts, by thresholding the classifier real-valued output, whether new cases of data will fall into one of two categories. The classifier used in the current analysis was based on a linear function of the form of ***w**^T^***x** + *b*, where *w* is the weight vector to be determined, x is the input vector representing EEG features and ***w**^T^***x** represents the dot product between the two vectors. It obtains the best model coefficients in *w* by minimizing the following regularized risk function:

$$\sum_{j=1}^{d}|w_{j}|+C\sum_{i=1}^{n}\varepsilon_{i}$$

where *d* represents the number of variables (i.e., EEG features) in total, *n* represents the number of records collected in the training set, and ε_*i*_ = max{0, 1 − *y_i_*(***w**^T^***x**_*i*_ + *b*)} denotes the so-called hinge loss to measure the training error [36], where *y_i_* represents the class label, such as “correct response” versus “incorrect response” of the record *i* that is numerically characterized by an input vector **x**_*i*_ (i.e., the vector of features extracted from that record).

A record consisting of 60 features of EEG data was extracted for each participant, including five frequency bands (theta 1, theta 2, alpha, beta, and gamma), three scalp locations (frontal, Fz; central, Cz; occipital, Oz), and four information processing stages (pre-stimulus baseline, encoding, retention, and retrieval). Features were binned separately based on trial accuracy and assigned a binary label indicating whether trials received correct (+1) or incorrect (−1) responses. Accordingly, EEG features receiving positive valence weightings can be interpreted as more highly predictive of correct trial performance, with those receiving negative valence predictive of incorrect performance. The SVM algorithm was applied in two models: (1) to classify correct vs. incorrect trial performance within each sample, referred to hereafter as *Model 1*, and (2) to classify between SZ and HC groups across correct and incorrect trials, referred to as *Model 2*.

Although the current analysis was based on a small study (12 HC and 40 SZ), a large number of EEG features (60) were used to represent each case. This circumstance poses risk for over-fitting, meaning that the resultant classifier could achieve good accuracy during training but poor validation accuracy. According to statistical learning theory [36], regularization is the most effective way to control over-fitting. SVM methods optimize a regularized loss function for the best classifier where either the two-norm regularizer ${\left\|w\right\|}^{2}=\sum_{j=1}^{d}w_{j}^{2}$ or one-norm regularizer $w_{1}=\sum_{j=1}^{d}|w_{j}|$ is used. In the current implementation, the 1-norm regularizer was chosen because this regularizer enforces sparsity of the weight vector *w*, meaning many entries of *w* will be zeros. More precisely, although 60 features were used in the SVM classifier training, when the classifier is built by SVM, only 3 ~ 10 features were actually used by the classifier because other features received zero weights in the model.

The parameter C in the 1-norm SVM was tuned in a 3-fold cross validation process where the respective data set was evenly split into 3 disjoint subsets. At each fold, we tested on a subset of the data the classifier obtained by SVM from the remaining data. Receiver operating characteristics (ROC) curves were used to examine the performance of the classifiers. Specifically, the area under the curve (AUC) was reported. We average the AUC values over the three folds for each choice of C in a range from 0.1 to 10 with a step size of 0.1. The value of C that produced the best cross validation performance was used to train the final classifier from all records. The cross validation performance for SVM with the chosen C value was also reported. In addition to AUC values, precision, recall, and F1 score were computed.

The analysis of *Model 2* presented an unbalance classification problem due to far fewer HCs (n = 12) than SZs (n = 40). Therefore, a commonly used procedure in SVM was adopted to balance the sample size. Specifically, the analysis penalized errors that occurred in the HC samples 3 times more than the errors in the SZ samples. This created the similar effect as up-sampling HC three times. Mathematically, this procedure corresponded to revising the regularized loss function as follows:

$$\sum_{j=1}^{d}|w_{j}|+C(3\sum_{i\in \{HN\}}\varepsilon_{i}+\sum_{i\in \{SZ\}}\varepsilon_{i})$$

## Results

### Demographics

Groups did not differ in basic demographic composition (Table 1). Three (15 %) participants in the HC sample were over age 55 (aged 63, 59, 56), while 8 (20 %) in the SZ sample were over 55 (aged 70, 66, 63, 62, 60, 59, 58, 58). Statistical analysis were unaffected by entering age as a covariate and reported results were not significantly changed by restricting the sample to those aged 55 or younger (N = 41).

### Behavioral data

Group comparison on SWMT behavioral data revealed overall lower accuracy in SZ than HC (Table 1). As a group, working memory performance was impaired in SZ participants based on MCCB WM composite score, but visual attention was within normal range based on the CPT-IP. SZ participants were estimated to have average range of IQ but, overall, scored lower than HC.

### Model 1: Classification of SWMT performance accuracy

#### Healthy normal sample

SVM Model 1 identified frontal (Fz) gamma activity during encoding and occipital (Oz) theta 2 during retrieval as the primary EEG features associated with SWMT accuracy in the HC sample (Table 2). Additional features retained in the model had weightings of .10 or less and were not regarded as meaningful for further analysis. The negative valence of feature weights indicated that higher values for each preceded incorrect behavioral responses. Model classification accuracy was 84 % and all additional performance statistics (F1 score = 0.96, precision = 0.92, recall = 1.0, estimated AUC of ROC = 0.98), suggested excellent model fit and stability. Cross-validation of this model applied to SZ data yielded lower, yet acceptable model, classification accuracy (74 %) and performance statistics (F1 score = 0.77, precision = 0.68, recall = 0.9, estimated AUC of ROC = 0.84). Accordingly, primary features determining SWMT performance in HC also applied to SZ; however, an overall decrease in model performance suggested that other or additional features were explanatory for SZ.

To further assess the stability of SVM Model 1 based on HC data, the analysis was repeated with features entered separately by stage of WM processing (i.e., baseline, encoding, retention, retrieval). This analysis was conducted to determine whether experimenter decisions regarding method of feature entry (i.e., 60 features entered simultaneously vs. 15 features entered into 4 separate models) would substantially influence the outcome of feature selection. Overall, the two approaches converged on the same primary features (Table 3). As observed with simultaneous entry of 60 EEG features, frontal gamma during encoding was the feature most highly weighted in predicting SWMT accuracy. Notably, the only two features identified at the encoding stage with non-zero weightings both involved gamma activity, the second feature being centrally distributed gamma, and together predicted SWMT trial performance with 96 % accuracy. Retrieval stage features also predicted SWMT with high accuracy (88 %) based primarily on occipital activity in gamma and theta 2 ranges (Table 3). In this case, the ordering of features differed slightly from the model constructed by simultaneous entry in that theta 2, rather than gamma, was most highly weighted. Furthermore, modeling data independently according to WM stage identified features that were evidently suppressed by the primary features of the original model. No feature representing the pre-trial baseline stage entered the original model when applied to HC data; however, a contribution of baseline activity accounted for almost entirely by central theta (feature weight = −1.13), in association with inaccurate performance, was identified when modeled independently. Finally, the contribution of retention stage activity to performance was best characterized by central theta 1, both when features were modeled simultaneously (Table 2, 3^rd^ ranked feature) and independently by WM stage.

#### Schizophrenia sample

Many more features entered the model when constructed using SZ sample data (Table 4), with central and frontal gamma during encoding identified as the primary classifiers of SWMT accuracy. As observed in the HC sample data, the valence of coefficients indicated that higher values for these features preceded incorrect behavioral responses. Interestingly, beta activity during retrieval was also identified as a predictor of trial accuracy but with a positive coefficient, indicating that higher activity preceded correct behavioral responses. Theta 1 during retention and theta 1 and gamma activity during retrieval entered as negative predictors of trial accuracy with weightings above .5. Overall classification accuracy was 80 % and model performance statistics (F1 score = 0.80, precision = 0.78, recall = 0.83, estimated AUC of ROC = 0.88) suggested good fit and stability. Importantly, although SVM modeled directly on SZ data performed slightly better than when parameters extracted from HC Model 1 were applied to SZ data (i.e., F1 scores of 0.80 and 0.77, respectively), gamma activity at encoding received the highest weightings in both cases.

### Model 2: Classification of diagnostic status

Features selected by SVM models used to classify diagnostic status (SZ labeled +1 and HC labeled −1) based on correct and incorrect behavioral responses are presented in Tables 5 and 6, respectively. Overall classification accuracy of 79 % was achieved by EEG features selected from correct response trials, with higher values of frontal and central theta at baseline associated with SZ group membership (Table 5). Gamma band activity during retrieval and encoding stages also entered the model but with relatively low weightings. Performance statistics of this diagnostic classification model were acceptable (F1 score = 0.87, precision = 0.77, recall = 1, estimated AUC of ROC = 0.77). SVM modeled on incorrect trials (Table 6) identified frontal alpha at retrieval as the highest weighted feature, with a near-zero contribution of central gamma during encoding. The valence of coefficients indicated that higher values were associated with HC group membership. Performance statistics of this diagnostic classification model using incorrect trial data were exactly identical to those of the other model using correct trial data. Taken together, these findings are interpreted to suggest that SZ is generally distinguished from HC by higher levels of low-frequency (theta 1) spectral power at pre-trial baseline, and lower levels of alpha band power during retrieval than HC, particularly when WM load exceeds capacity (i.e., incorrect responses).

### Concurrent and external validity

#### SVM Model 1

**Fig. 2:**
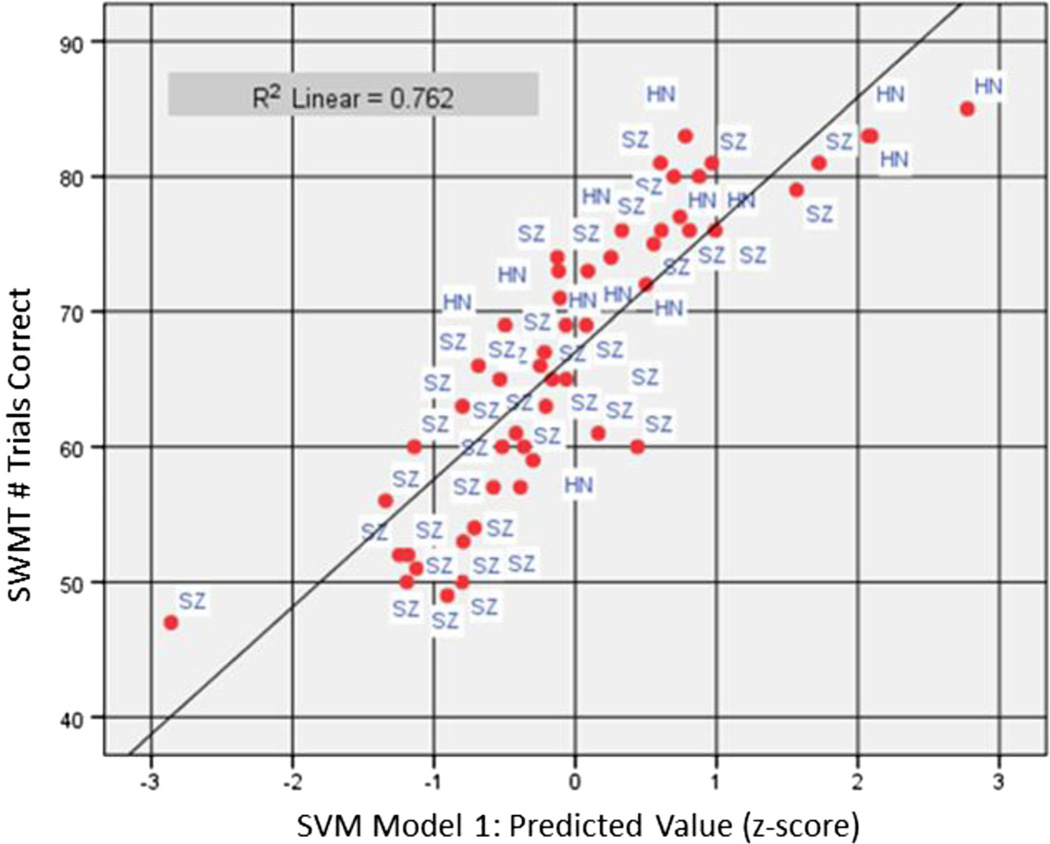
Scatterplot of Sternberg Working Memory Task (SWMT) performance (out of 90 trials possible) as predicted by SVM Model 1 across the full study sample (*N* = 52). Multiple regression explained 76 % of the variance in SWMT performance based on frontal gamma activity during encoding and central theta 1 activity during retention. Both correct and incorrect trials entered the model for each feature. SVM Model 1 score (x-axis) represents the residual difference between predicted (trend line) and observed value for SWMT performance

As a test of concurrent validity based on classification method, EEG features selected by SVM Model 1 in HC data (Table 2) were submitted to stepwise linear regression as predictors of SWMT total score in the full sample of HC and SZ participants (*N* = 52). The model was highly statistically significant (*F*_(4, 47)_ = 37.67, *p* < 0.0005, *R* = 0.87) and explained 76 % of the variance in SWMT score (Fig. 2). Central theta 1 during retention in correct trials entered as the first step, frontal gamma during encoding in correct trials as the second step, frontal gamma during encoding in incorrect trials as the third step, and central theta 1 during retention in incorrect trials as the fourth and final step (Table 7). Beta and partial correlation coefficients suggested that when participants answered incorrectly, presumably challenged by higher WM load, performance was associated with higher levels of frontal gamma during encoding and central theta 1 power during retention (beta = 0.38 and 0.39, partial *r* = 0.51 and 0.48, respectively), while lower levels were associated with correct responses (beta = −0.44 and −0.55, partial *r* = −0.62 and −0.61, respectively). The same EEG features were retained, with exactly the same model coefficients, when the regression analysis was repeated by replacing the predictors with the 1^st^ ranked feature of each WM stage (Table 3).

**Fig. 3:**
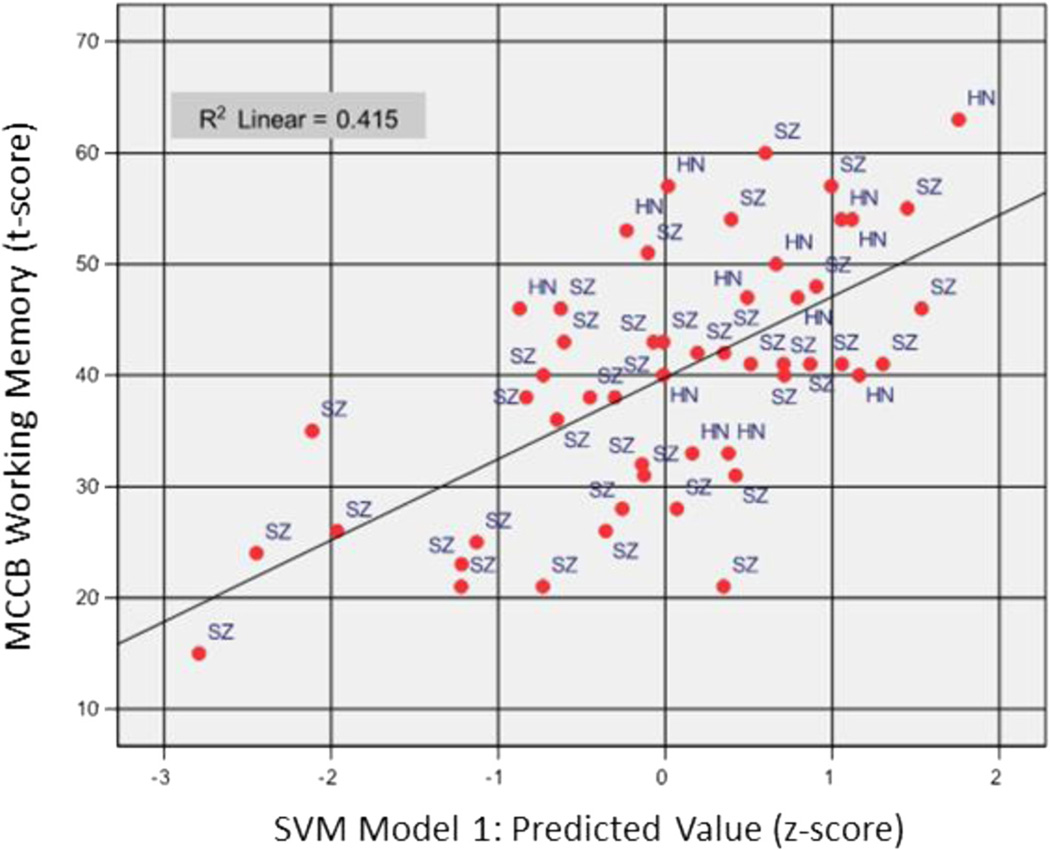
Scatterplot of MCCB Working Memory (WM) Composite score (standardized; t-score) as predicted by SVM Model 1 across the full study sample (*N* = 52). Multiple regression explained 42 % of the variance in MCCB WM score based on frontal gamma activity during encoding and central theta 1 activity during retention, with only data from correct trials entered entering the model for each feature. SVM Model 1 score (x-axis) represents the residual difference between predicted (trend line) and observed value for MCCB WM score

**Fig. 4:**
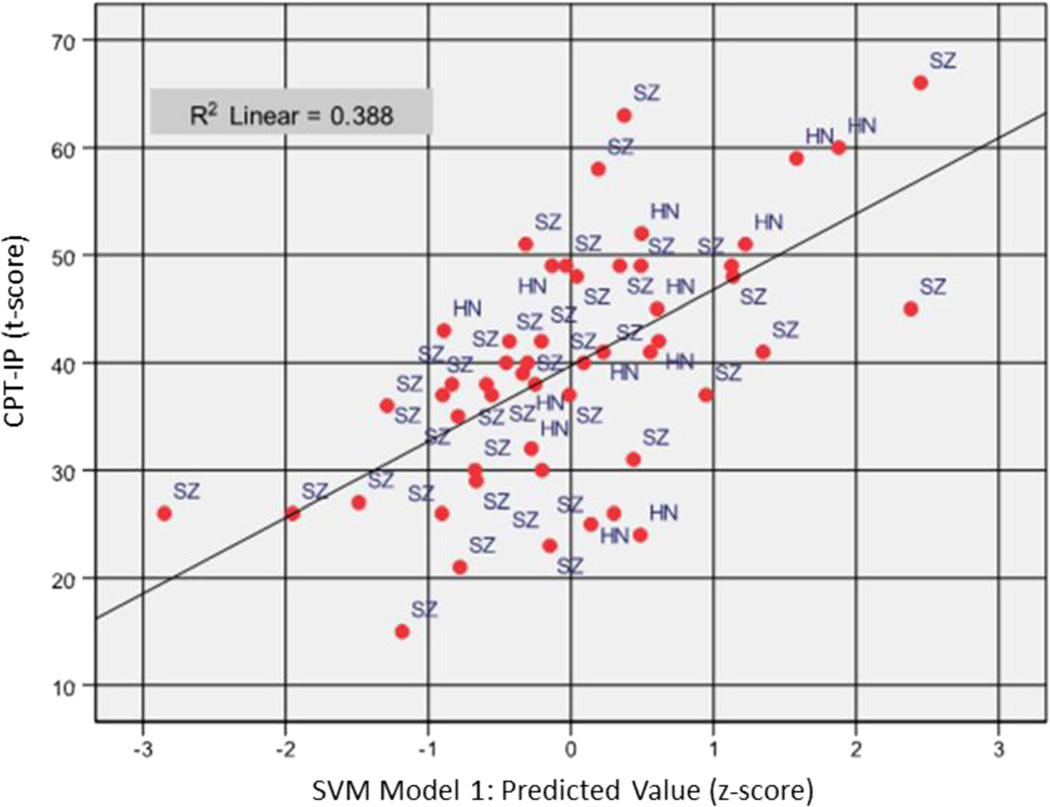
Scatterplot of Continuous Performance Test-Identical Pairs version (CPT-IP) score (standardized; t-score) as predicted by SVM Model 1 across the full study sample (*N* = 52). Multiple regression explained 39 % of the variance in CPT-IP score based on frontal gamma activity during encoding and central theta 1 activity during retention for correct trials and occipital gamma activity at retrieval for incorrect trials. SVM Model 1 score (x-axis) represents the residual difference between predicted (trend line) and observed value for CPT-IP score

To examine external validity of the EEG features derived by SVM, the same regression model was repeated to predict MCCB WM Composite (Fig. 3) and CPT-IP (Fig. 4) scores in separate analysis. MCCB WM Composite score was predicted (*F*_(2, 49)_ = 17.39, *p* < 0.0005, *R* = 0.64) with 42 % of variance explained by two features, i.e., frontal gamma during encoding in correct trials (*R*^2^ = 0.31, F change_(1, 50)_ = 23.92, significant F change < 0.0005) and central theta 1 during retention in correct trials (*R*^2^ = 0.42, *R*^2^ change = 0.09, F change_(1, 49)_ = 7.67, significant F change = 0.008). The direction of association was consistent with previous models, with frontal gamma at encoding (beta = −0.59, partial *r* = −0.61) and central theta 1 at retention (beta = −0.30, partial *r* = −0.37) associated negatively with working memory test performance. The CPT-IP was selected as an additional cross-validation measure due to dependence of this task on visual encoding and retrieval processes similar to the SWMT. CPT-IP performance was predicted with 39 % of variance explained (*F*_(3, 48)_ = 10.15, *p* < 0.0005, *R* = 0.62) based on three features: frontal gamma activity at encoding in correct trials as the first step (*R*^2^= 0.19, F change_(1, 50)_ = 11.54, significant F change = 0.001), central theta 1 activity at retention in correct trials as the second step (*R*^2^ = 30, *R*^2^ change = 0.11, F change_(1, 49)_ = 7.90, significant F change = 0.007), and occipital gamma activity at retrieval in incorrect trials as the third step (*R*^2^= 39, *R*^2^ change = 0.09, F change_(1, 48)_ = 6.89, significant F change = 0.012). Consistent with prior models, beta and partial correlations for frontal gamma during encoding and central delta during retention in correct trials were negatively associated with CPT-IP AGT (beta = −0.45 and −0.32, partial *r* = −0.50 and −0.37, respectively) while occipital gamma during retrieval in incorrect trials entered with positive coefficients (beta = 0.30, partial correlation = 0.35). These results confirmed that EEG features modeled on SWMT performance are generalizable with respect to neuropsychological measures of working memory and visual attention.

#### SVM Model 2

To cross-validate the diagnostic classification accuracy of SVM Model 2, derived features (Tables 5 and 6), were submitted to discriminant function analyses of diagnostic membership (i.e., HC vs. SZ) using stepwise entry. The overall Wilk's lambda, Λ = 0.59, χ^2^(df = 4) = 25.67, *p* < 0.0001, indicated that there was a significant group-wise difference by diagnosis across four retained EEG features, with group centroids of 1.43 and −.48 for HC and SZ, respectively. The correlation structure of the discriminant function (Table 8) indicates that HC was classified with higher frontal alpha at retrieval and central gamma at encoding on incorrect trials, while SZ was associated with higher frontal theta 1 at baseline and central gamma at retrieval on correct trials. Overall diagnostic classification accuracy in the full sample was 87 % (sensitivity 90 %, specificity 77 %) with positive predicative power (SZ diagnosis) of 92 % and negative predictive power of 71 % probability. Leave-one-out cross-validation of this model replicated classifications with 83 % accuracy.

## Discussion

The primary aim of the current study was to evaluate the utility of machine learning methodology, specifically SVM, as a novel approach of EEG feature selection. EEG data involves many more variables than can be feasibility evaluated using conventional between-groups statistical contrasts, a problem that requires experimenter decisions guiding *a priori* selection of features submitted to hypothesis testing. In doing so, questions remain as to whether the selected features, among an extensive range of possibilities, are indeed those most critical to the questions asked of the data. Machine learning approaches, in contrast, offer the benefit of considering all data and empirically determining the most relevant features from all possible solutions. In this way, machine learning solutions represent a paradigm shift from rationally deductive to data inductive methodology.

The current study employed machine learning classification to identify (1) EEG features predictive of SWMT accuracy in healthy adults, (2) EEG features predictive of SWMT accuracy in schizophrenia, and (3) controlling for SWMT accuracy, EEG features that distinguished healthy from schizophrenia group status. Using 1-norm SVM classification and 60 features based on SWMT stage (4; baseline, encode, retain, retrieve), EEG frequency band (5; theta 1, theta 2, alpha, beta, gamma), and electrode site (3; Fz, Cz, Oz), frontal gamma-band activity at encoding was identified as the primary classifier of trial accuracy (Tables 2 and 3), while frontal-central gamma also contributed substantially to classifiers constructed by diagnostic status (Table 8). In addition, the level of low-frequency activity during the pre-stimulus baseline and activation of alpha during memory retrieval were identified as important diagnostic differences (Tables 5 and 8). In each case model performance was assessed by cross-validation and determined to adequately fit the data based on several metrics (i.e., F1-score, precision, recall, and estimated area under the ROC curve). Importantly, the EEG features identified by SVM seem both plausible and generalizable given prior literature reports regarding the role of gamma and alpha activity in working memory function and commonly higher levels of low frequency activity in resting EEG of individuals with schizophrenia.

**Fig. 5:**
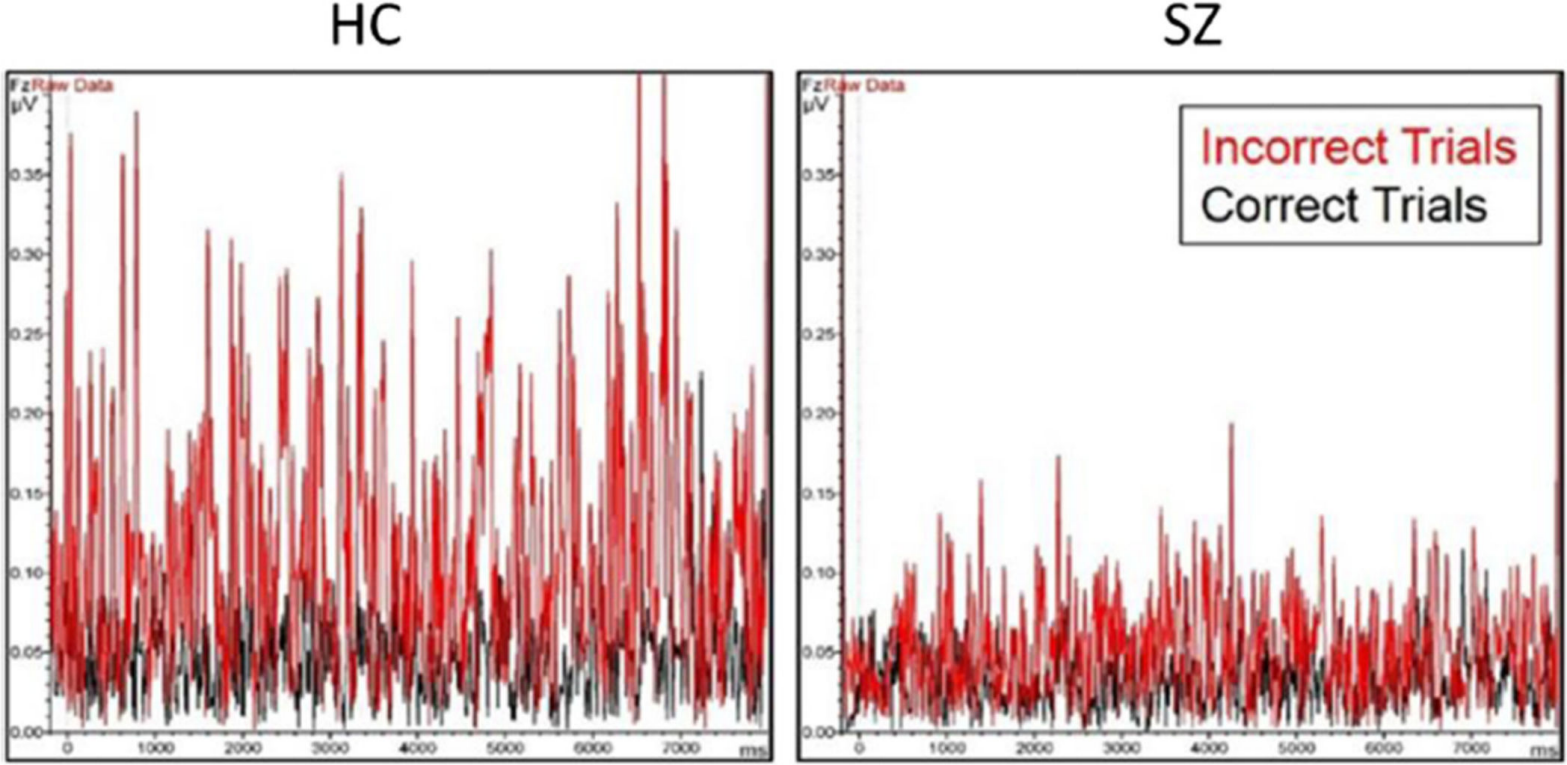
Overlay of group average data for correct and incorrect trials extracted in gamma band (31.48 – 49.16 Hz) during encoding stage. Gamma increased significantly preceding incorrect relative to correct trials for both groups (paired-samples t tests; HC, *t*(11) = 5.37, *p* < 0.0005; SZ, *t*(39) = 7.01, *p* < 0.0005) and interacted by group (Wilk's Λ = 0.86, *F*_(1, 50)_ = 8.46, *p* = 0.005), with HC evidencing significantly greater range of modulation by accuracy level. Data submitted to statistical analysis was extracted from 1000–7000 ms, essentially containing the period of 200 ms prior to onset of first stimuli in set to 200 ms following onset of the 5^th^ stimuli of the memory set (or 1400 ms following offset of the 4^th^ stimuli). Importantly, as depicted in the figure, differences in gamma activity by accuracy were present before onset of first stimuli of each trial (0–1200 ms) and, therefore, are not interpreted to represent a memory load effect in these data

Published reports describe an upward modulation of gamma band activity in response to higher working memory load in healthy participants, and an overall attenuation of gamma with a failure to modulate at higher memory loads in schizophrenia [24]. Our data partially support this finding but with an important difference in interpretation. As shown in Fig. 5, significant upward modulation of frontal gamma power in *incorrect* relative to *correct* trials is evident in schizophrenia and healthy samples alike. When tested statistically, encoding gamma was found to be significantly increased in incorrect relative to correct trials for both groups (paired-samples t tests; HC, *t*(11) = 5.37, *p* < 0.0005; SZ, *t*(39) = 7.01, *p* < 0.0005); however, the strength of this upward modulation was significantly greater in healthy participants (Correct-Incorrect×Group interaction effect, Wilk's Λ = 0.86, *F*_(1, 50)_ = 8.46, *p* = 0.005). However, of note, gamma modulation with accuracy appears to be evident by the time the first stimulus of the memory set is presented (i.e., by 1200 ms). Therefore, current results are not interpreted purely in context of a memory load effect. Rather, given that differences in gamma preceding correct and incorrect trials are already present and persist in the early stage of encoding, elevations of gamma band power may reflect changes in cognitive preparedness that occur from trial to trial. This interpretation is not entirely inconsistent with prior findings associating upward modulation of gamma at increased memory load with better working memory function. We suggest that the early presence of increased gamma preceding incorrect trials could indicate that gamma has already elevated to peak level, limiting the ability to further increase gamma with encoding of new information and, thereby, reducing trial accuracy. Further examination of reasons for elevated gamma preceding incorrect trials is beyond the scope of the current analysis, but possible explanations could include the extended maintenance of information, or perhaps cognitive response to error feedback, from the preceding trial. Pertinent to the current analysis, it would appear that individual differences in the overall magnitude of encoding gamma, found to be greater in healthy than schizophrenia groups, is better represented in incorrect than correct trials and, for this reason, activity preceding incorrect trials was found most predictive of SWMT performance in both groups. Consistent with prior findings [24, 25], it does appear that working memory impairment in schizophrenia relates, in part, to a restriction of range in the ability to upregulate gamma in response to cognitive challenge.

Furthermore, SVM also identified central gamma activity during encoding as the most highly weighted feature predicting SWMT performance in schizophrenia, suggesting gamma activity extended over greater cortical areas than in healthy participants. Studies of postmortem brain tissue have provided strong evidence that the GABAergic system of left DLPFC is impaired in schizophrenia [37–39]. GABAergic interneurons appear to be crucial elements in the generation of synchronous neuronal activity in the gamma band [40–44]. Results of phase locking and coherence analyses in schizophrenia patients further suggest that neuronal network functioning is impaired due to a failure of neuronal synchrony at gamma band frequency [45]. Based on present and previous studies, we speculate that extension of gamma activity from frontal to central cortical areas in patients may be compensatory in response to inefficiency of frontal activity generated in the DLPFC [38, 46].

Data-driven approaches for EEG feature selection would seem particularly useful, if not essential, when working with complex cognitive experiments that entail several stages of information processing, as well as for common experiments that can nonetheless be analyzed by spectral decomposition of EEG in multi-channel recordings. Although 1-norm SVM classification was selected for the current study, this is by no means the only approach to consider and research in this area could be expanded by comparing and optimizing other machine learning approaches for use with EEG data. Graphical models, which take into account some network correlations, or Gaussian Process regression, which can identify nonlinear relationships in the data, may be other promising approaches. A future direction for analysis based on SVM is to develop new machine learning methods that can optimize simultaneous modeling of the spatial and temporal distribution of the EEG features, to better account for change in EEG frequency amplitude at different scalp locations over time. Feature selection using such spatial-temporal modeling could also become more precise by accommodating single-trial data and larger electrode arrays.

The current study was limited in terms of sample size, particularly of healthy community participants and, therefore, models derived may not be optimized for the broad range of abilities represented in the population at large. Our objective in analyzing the current data set was primarily to demonstrate how SVM could be applied to data analytic questions that involve many potential dependent measures. As an analytic solution for big data problems, the performance of SVM improves with larger and, presumably, more stable datasets inclusive of the full range of possible values on the parameters involved. Nevertheless, cross-validation of the features selected by SVM with regard to external measures of working memory performance and diagnostic classification by discriminant function suggested that the derived models performed well within the constraints of the current sample. EEG data modeled on SWMT performance in healthy participants explained 76 % of variance in task performance across samples and demonstrated a linear relationship that appeared a good fit for schizophrenia data over the full range of performance (Fig. 2). Of note, healthy participants generally responded to at least 70 of 90 trials correctly, representing 78 % accuracy. Individuals with schizophrenia who performed in this range also exhibited neural activity in the average to above-average range (i.e., standard score values of 0 and above) relative to the sample distribution, while EEG values were generally within 1 standard deviation below average for those performing below 78 % accuracy. With larger samples, contributing to better overall normative estimates, it would be conceivable to construct neurophysiological test batteries comparable to standard neuropsychological tests that provide individual measures of performance on multiple domains based on precise measures of neural activity. This information could inform treatment selection and outcome measurement of interventions targeting cognitive impairment through cognitive remediation training using task-related neurofeedback methods [47, 48].

## Conclusions

In summary, we conclude that SVM successfully identified EEG features associated with working memory performance that are consistent with, and rationally predicted, based on prior literature. Selected features highlight the roles of gamma activity during encoding and theta during memory retention as EEG components contributing similarly to Sternberg performance in both healthy and schizophrenia study samples. Importantly, these same features explained substantial portions of variance in working memory and visual attention ability when assessed by standardized neuropsychological tests, lending support to the external validity of these findings. Furthermore, SVM produced a diagnostic classifier achieving 87 % accuracy in distinguishing individuals with schizophrenia. Gamma activity during encoding remained to be a primary feature distinguishing groups, with lower alpha during retrieval and increased theta during pre-stimulus baseline as additional features characterizing schizophrenia. These results, based only on data collected using the Sternberg task, compare favorably with another recent example of SVM applied to P300 and mismatch negativity task data, where nearly 85 % classification accuracy was achieved [49], as well as to prior efforts to enhance diagnostic classification using multiple EEG experiments and traditional regression approaches [50]. Taken together, machine learning approaches, such as SVM, show considerable potential as an analytic strategy for data reduction and feature selection of complex EEG datasets.

## Figures and Tables

**Table 1:** Sample descriptive statistics

	SZ (*n*=40)		HC (*n*=12)			
				
Variable	Mean	(SD)	Mean	(SD)	T-score (df=50)	*p*-value^b^
Age	46.10	12.41	43.33	13.22	0.67	0.510
Age of Onset	20.84	6.70	-	-		
Hospitalizations (#)	13.77	25.08	-	-		
PANSS total	56.87	13.22	-	-		
Positive	16.20	5.80	-	-		
Negative	13.07	4.78	-	-		
General	27.60	7.18	-	-		
Antipsychotic CPZ Eq	578.81	404.73	-	-		
Antipsychotic Any	37 of 40	-	-	-		
Traditional only	9 of 37	-	-	-		
Atypical only	25 of 37	-	-	-		
SWMT total	63.33	11.02	75.58	7.62	3.59	0.001
WTAR FSIQ	91.45	13.98	100.73	15.82	1.90	0.064
CPT-IPa	38.03	11.61	42.75	11.66	1.24	0.220
MCCB WM Composite^a^	37.18	10.80	46.67	8.99	2.77	0.008
	%		%		c2 (df=1)	*p*-value^b^
Gender (Male)	57.50	-	50.00	-	0.21	0.646
Race (Caucasian)	35.00	-	58.00	-	1.23	0.267
Handedness (Right)	85.00	-	100.00	-	2.04	0.362

PANSS: Positive and Negative Syndrome Scale, CPZ Eq: chlorpromazine equivalent, SWMT: Sternberg Working Memory Task, WTAR: Wechsler Test of Adult Reading, CPT-IP: Continuous Performance Test-Identical Pairs, MCCB: MATRICS Cognitive Composite Battery

a Age, education, and gender corrected t-scores reported according to MCCB normative sample

b Statistic reported based on two-tailed test

**Table 2:** EEG Features Predicting Trial Accuracy in HC

Location	WM stage	Frequency	Feature Weight
Frontal	Encode	gamma	−1.500
Occipital	Retrieve	theta 2	−0.861
Central	Retain	theta 1	−0.097
Central	Retrieve	gamma	−0.096
Occipital	Baseline	theta 1	−0.035

Features extracted by 1-norm SVM to classify correct vs. incorrect trials in HN. Model based on simultaneous entry of 60 EEG features with correct trials labeled 1 and incorrect labeled −1. All other features weighted at 0

**Table 3:** SVM Model 1 Coefficients Extracted by Stage of Working Memory

WM Stage	Frequency	Feature Weight
Baseline		Accuracy = .77
Central	theta 2	−1.127
Occipital	beta	−0.183
Frontal	theta 1	−0.158
Occipital	theta 2	−0.143
	intercept	0.480
Encode		Accuracy = .96
Frontal	gamma	−1.693
Central	gamma	−0.748
	intercept	0.607
Retain		Accuracy = .77
Central	theta 1	−1.192
Occipital	theta 2	−0.307
	intercept	0.500
Retrieve		Accuracy = .88
Occipital	gamma	−1.380
Occipital	theta 2	−0.646
Central	gamma	−0.261
	intercept	0.597

Four separate SVM Models were constructed in HN with entry of 60 EEG Features by WM Stage. Features extracted by 1-norm SVM to classify correct vs. incorrect trials with correct trials labeled 1 and incorrect labeled −1. Intercepts of four models were equivalent. All other features weighted at 0

**Table 4:** EEG Features Predicting Trial Accuracy in SZ

Location	WM stage	Frequency	Feature Weight
Central	Encode	gamma	−1.169
Frontal	Encode	gamma	−0.916
Central	Retrieve	beta	0.704
Central	Retain	theta 1	−0.611
Central	Retrieve	theta 1	−0.601
Frontal	Retrieve	gamma	−0.600
Central	Encode	alpha	0.409
Occipital	Retrieve	theta 1	−0.371
Occipital	Retain	beta	−0.350
Central	Baseline	theta 1	−0.204
Frontal	Retain	theta 1	−0.168
Frontal	Encode	theta 1	−0.032
Frontal	Encode	theta 2	−0.029
Occipital	Retain	gamma	−0.001

Features extracted by 1-norm SVM to classify correct vs. incorrect trials in SZ. Model based on simultaneous entry of 60 EEG features with correct trials labeled 1 and incorrect labeled −1. All other features weighted at 0

**Table 5:** EEG Features Predicting Diagnostic Group Based on Correct Trials

Feature Name	WM stage	Frequency	Feature Weight
Frontal	Baseline	theta 1	0.529
Central	Baseline	theta 2	0.302
Central	Retrieve	gamma	0.254
Frontal	Encode	gamma	0.108
Frontal	Baseline	theta 2	0.037

Features extracted by 1-norm SVM to classify HN vs. SZ status in correct trial data. SZ is labeled 1 and HN labeled −1. All other features weighted at 0

**Table 6:** EEG Features Predicting Diagnostic Group Based on Incorrect Trials

Location	WM Stage	Frequency	Feature Weight
Frontal	Retrieve	alpha	−0.541
Central	Encode	gamma	−0.027

Features extracted by 1-norm SVM to classify HN vs. SZ status in incorrect trial data. SZ is labeled 1 and HN labeled −1. All other features weighted at 0

**Table 7:** Multiple Linear Regression Model Predicting SWMT Performance by SVM Model 1 Features

Step	R	R^2^	Std. Error	Δ R^2^	Δ *F*	Δ *F p value*
1	.450^a^	.202	9.740	.202	12.661	.001
2	.655^b^	.428	8.327	.226	19.410	.000
3	.832^c^	.692	6.177	.263	41.051	.000
4	.873^d^	.762	5.484	.070	13.894	.001

Multiple linear regression based on all SVM Model 1 features achieved maximum fit based on frontal gamma during encoding and central theta 1 during retention. Features selected using forward-stepwise entry in the following order: ^1^Central Retain Theta 1 – correct; ^2^(1) + Frontal Encode Gamma – correct; ^3^(1, 2) + Frontal Encode Gamma – incorrect, ^4^Predictors (1, 2, 3) + Central Retain Theta 1 – incorrect

**Table 8:** Discriminant Function Structure Matrix

Location	WM Stage	Accuracy	Frequency	Function
Frontal	Retrieve	incorrect	alpha	0.571
Central	Encode	incorrect	gamma	0.536
Frontal	Baseline	correct	theta 1	−0.473
Central	Retrieve	correct	gamma	−0.340
Central^a^	Baseline	correct	theta 2	−0.325
Frontal^a^	Baseline	correct	theta 2	−0.219
Frontal^a^	Encode	correct	gamma	−0.003

Pooled within-group correlations between SVM Model 2 features and standardized canonical discriminant functions classifying SZ vs. HC.

a Feature excluded from analysis by stepwise entry
